# Unraveling the relationship among inflammatory responses, oxidative damage, and host susceptibility to *Opisthorchis viverrini* infection: A comparative analysis in animal models

**DOI:** 10.14202/vetworld.2023.2303-2312

**Published:** 2023-11-19

**Authors:** Sirikachorn Tangkawattana, Watcharapol Suyapoh, Nathamon Taiki, Paramin Tookampee, Ravisara Chitchak, Theerayut Thongrin, Prasarn Tangkawattana

**Affiliations:** 1 Department of Veterinary Pathobiology, Faculty of Veterinary Medicine, Khon Kaen University, Khon Kaen, Thailand; 2 WHO Collaborating Centre for Research and Control of Opisthorchiasis (Southeast Asian Liver Fluke Disease), Tropical Disease Research Center, Khon Kaen University, Khon Kaen, Thailand; 3 Department of Veterinary Science, Faculty of Veterinary Science, Prince of Songkla University, Songkhla, Thailand; 4 Doctor of Veterinary Medicine Program, Faculty of Veterinary Medicine, Khon Kaen University, Thailand; 5 Master of Science Program in Veterinary Science, Faculty of Veterinary Medicine, Khon Kaen University, Thailand

**Keywords:** cholangiocarcinoma, DNA damage, inflammatory reactions, Syrian golden hamsters

## Abstract

**Background and Aim:**

*Opisthorchis viverrini* infection-induced inflammation contributes to cholangiocarcinoma (CCA) development in humans and animals. Inflammation generates free radicals, such as reactive oxygen species and reactive nitrogen species (RNS), which damage the host’s DNA. However, only 5% of *O. viverrini*-infected individuals develop malignancy, suggesting that variations in the inflammatory response of individuals to the parasite may influence susceptibility. Due to limitations in studying human susceptibility, we used an animal model to investigate the profiles of inflammatory reactions, oxidative burst, and irreversible DNA damage. This study aimed to explore the potential role of inflammation and RNS in causing DNA damage that may predispose susceptible hosts and non-susceptible animal models to cancer development in *O. viverrini* infection.

**Materials and Methods:**

This experimental study was conducted on 30 Syrian golden hamsters (OV-H) and 30 BALB/c mice (OV-M) infected with *O. viverrini*, representing susceptible and non-susceptible models, respectively. Five animals per group were examined at six predetermined time points during the experiment. Biliary tract samples were systematically investigated using histopathological evaluation for inflammatory cell infiltration and immunohistochemical staining for RNS production and markers of DNA damage, including nitrotyrosine and 8-hydroxy-2′-deoxyguanosine. These features were quantified and compared among the experimental groups. Mann–Whitney U-test was used for statistical analysis, with p < 0.05 considered statistically significant.

**Results:**

The comparison revealed that the OV-M group exhibited significantly earlier and higher rates of inflammatory cell infiltration during the acute phase, whereas the OV-H group exhibited chronic and more severe inflammation (p < 0.020). Intracellular RNS production and DNA damage were closely associated with the inflammatory response.

**Conclusion:**

This study demonstrates differential responses in susceptible and non-susceptible models of *O. viverrini* infection regarding disease onset and duration, as well as intracellular RNS production and DNA damage caused by inflammation. Persistent inflammation generated oxidatively damaged DNA, which is a distinct pathological characteristic of susceptible hosts and may be critical for CCA development.

## Introduction

Opisthorchiasis, a chronic infection caused by the liver fluke *Opisthorchis viverrini*, is a significant public health concern in countries along the Greater Mekong Subregion, including Lao People’s Democratic Republic, Cambodia, Vietnam, and Thailand. This parasitic infection is a crucial risk factor for cholangiocarcinoma (CCA) development [1–3]. However, of the more than 12 million people infected with *O. viverrini* [2], only approximately 5% develop malignancy [4–6]. Since sequential studies in humans are challenging due to ethical considerations, animal models are used for investigative purposes. Animal models have been employed to study host susceptibility to foodborne species of *Opisthorchis* [7]. The ability of rodent models, such as mice and hamsters, to clear liver fluke infection and resist their establishment has been assessed, and worms have also been recovered. BALB/c mice can eliminate and resist liver fluke infections, whereas hamsters are not [8–11]. However, the inflammatory patterns and their pathological roles in parasitic helminth infection, particularly *O. viverrini*, in susceptible and non-susceptible hosts remain unelucidated.

Chronic inflammation is a pivotal factor in CCA development [12]. *Opisthorchis viverrini* infection triggers persistent inflammation through biological, chemical, and physical pathways [13, 14], which activate various inflammatory cells, resulting in the production of reactive oxygen species (ROS) and reactive nitrogen species (RNS) [15]. These free radicals are significantly involved in causing DNA damage and inhibiting DNA repair enzymes, thereby predisposing individuals to CCA development [16, 17].

The accumulation of DNA damage from prolonged, chronic inflammation due to *O. viverrini* infection leads to the neoplastic transformation of cholangiocytes and subsequent CCA development. This experimental study compared the differences in inflammatory responses and the resultant generation of RNS and DNA damage caused by *O. viverrini* infection in susceptible (hamsters) and non-susceptible (BALB/c mice) host models using histopathological assessment and immunohistochemical (IHC) analysis.

## Materials and Methods

### Ethical approval

All animals were housed and maintained under controlled standard conditions at the Laboratory Animal Care Unit, Faculty of Medicine and the Northeast Laboratory Animal Center, Khon Kaen University. Ethical approval for the study was obtained from the Animal Ethics Committee of Khon Kaen University (IACUC-KKU 78/2561).

### Study period and location

The study was conducted on the archived tissue blocks (prepared during 2019-2020) from April to September 2021 in the Tropical Disease Research, Faculty of Medicine, Khon Kaen University, Thailand.

### Preparation of infective-stage metacercaria

*Opisthorchis viverrini* metacercaria were obtained from freshwater cyprinoid fish collected from an endemic area in Thailand. Briefly, 50 kg of fresh fish was minced and subjected to digestion using a synthetic enzyme solution containing 0.25% pepsin and 0.15% hydrochloric acid in a water bath at 37°C for 1 h. Then, the suspension was filtered through sieves of varying pore sizes to collect metacercariae from the resultant filtrates. Finally, the collected metacercariae were precipitated in normal saline [18] and morphologically identified [19].

### Experimental design and sample collection

Thirty male immunodeficient inbred mice (BALB/c; OV-M group) and 30 male Syrian golden hamsters (*Mesocricetus auratus*; OV-H group) aged 6–8 weeks were infected with *O. viverrini* by feeding 50 metacercariae to each animal through intragastric intubation.

**Figure-1:**
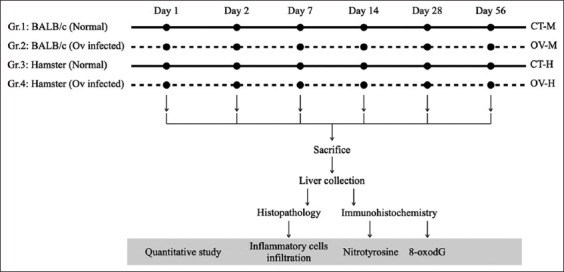
Experimental design flowchart. Five animals of each group from susceptible and non-susceptible animal models (BALB/c mice and hamsters) infected with *Opisthorchis viverrini* were sacrificed and collected the liver samples at 1, 2, 7, 14, 28, and 56 days after infection. Then, they were processed for histopathological and immunohistochemical evaluations. OV-M = BALB/c mice with *O. viverrini* infection, OV-H = Syrian golden hamsters with *O. viverrini* infection.

Five individuals each from the OV-H and OV-M groups were euthanized through isoflurane inhalation at 1, 2, 7, 14, 28, and 56 days post-infection (DPI) (Table-1). The livers were removed for use in histopathological and IHC studies (Figure-1).

**Table-1:** Experimental groups of BALB/c mice and hamsters infected with *O. viverrini*.

Groups	*O. viverrini* infected	Day sacrificing number					

		D1	D2	D7	D14	D28	D56
OV-M	+	5	5	5	5	5	5
OV-H	+	5	5	5	5	5	5

Five mice and hamsters of each group were sacrificed on a designated time point. *O. viverrini=Opisthorchis viverrini*

### Quantification of inflammatory cell infiltrates

The infiltration of inflammatory cells in the liver and biliary tree, including the common bile duct (CBD) and intrahepatic bile duct (IHB), was quantified. Briefly, after fixing and processing the liver specimens into paraffin blocks using a standard histological technique, serial sections (4 μm) were cut and stained with hematoxylin and eosin. Inflammatory cells were identified and evaluated according to Dulaimi *et al*. [20]. The infiltrating leukocytes in the biliary tract were quantified by analyzing 10 high-power fields under light microscopy [21].

### Immunohistochemical staining of nitrotyrosine and 8-hydroxy-2′-deoxyguanosine (8-oxo-dG)

Immunohistochemical staining was performed on the sections of hepatic tissues. Briefly, the sections were pretreated in an oven at 60°C for 15 min, deparaffinized in xylene, and rehydrated in a descending series of alcohol solutions. Antigen retrieval was performed using citrate buffer (pH = 6.0) in a high-pressure cooker for 10 min. Then, endogenous peroxidase activity was blocked using 5% hydrogen peroxide (H_2_O_2_), followed by blocking of non-specific binding by incubating the histological sections with 1% normal horse serum in phosphate-buffered saline for 45 min at room temperature. Reactive nitrogen species production was assessed by incubating the sections overnight with polyclonal rabbit anti-nitrotyrosine antibody (A21285, 1:50; Thermo Fisher Scientific, USA), followed by staining using a DAKO ChemMate Envision Kit (Dako, Glostrup, Denmark). DNA damage responses were evaluated by incubating the slides overnight with polyclonal goat anti-8-oxo-dG antibody (AB5830, 1:200; Merck, NJ, USA). Then, the slides were washed and incubated with polyclonal anti-goat IgG-HRP conjugate (1:300; Abcam, Cambridge, UK) for immunoperoxidase staining. Next, the color was intensified using 3,3′-diaminobenzidine in 0.003% H_2_O_2_ and counterstained with Mayer hematoxylin. Finally, the tissues were dehydrated, cleared, and mounted. The positively stained cells for each marker were quantified by manually counting the number of positive cells/500 biliary cells within the microscopic field [22, 23]. The proportion of IHC-stained cells was calculated and compared between groups.

### Statistical analysis

All data were statistically analyzed using statistical package for the social sciences v23.0 (IBM Corp., NY, USA). Differences between two groups were evaluated using the Mann–Whitney U-test. p < 0.05 was considered statistically significant.

## Results

Sections of liver and biliary trees from a total of 60 hamsters and mice infected with *O. viverrini* were histopathologically examined to study the host tissue immune response. The investigation focused on the presence of inflammatory cell infiltration as an indicator of the immune response. In addition, IHC was performed to detect increases in ROS and DNA damage in biliary cells using nitrotyrosine and 8-oxo-dG as markers, respectively.

### Pattern of inflammatory cell infiltration in susceptible and non-susceptible hosts

**Figure-2:**
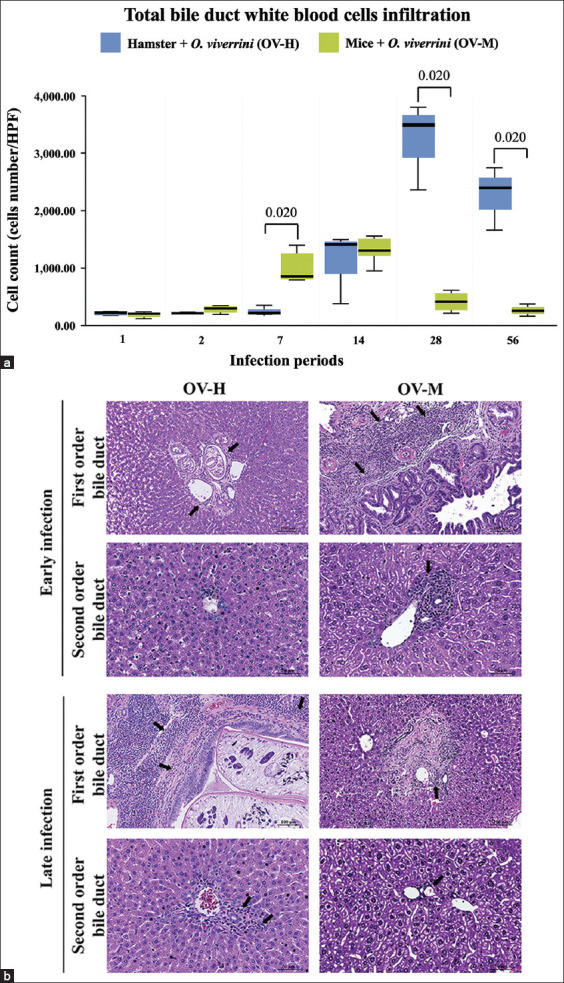
A comparison of white blood cells infiltration at the biliary areas of the hamsters and mice infected with *Opisthorchis viverrini* from 1 to 56 days. (a) Statistical analysis revealed a significant difference in the cell numbers of infiltrating leukocytes between the two animal models (p < 0.05). (b) Representative photomicrograph illustrated different inflammatory response at 1^st^ and 2^nd^ order of IHB in *O. viverrini*-infected hamster (OV-H), and *O. viverrini* infected BALB/c mice (OV-M). The arrows indicate the inflammatory cell infiltration at peribiliary area (hematoxylin and eosin, original magnification, first-order bile duct = ×20, scale bar depicts 100 μm; second-order bile duct = ×40, scale bar depicts 50 μm).

To investigate the pattern of inflammatory responses in the bile ducts of hamsters and mice infected with *O. viverrini*, we quantitatively evaluated the infiltrative leukocytes in hepatic tissues, including the CBD and IHB, from the OV-H and OV-M groups at six predetermined time points following infection (Figure-2a). In the OV-M group, the number of inflammatory cells was higher during the early phase compared with the number of the inflammatory cells in the OV-H group (Figure-2b). The major populations of infiltrating leukocytes comprised neutrophils, lymphocytes, eosinophils, and macrophages. Infiltration was predominantly observed in peri-bile duct tissue around the CBD and IHB. A small number of inflammatory cells were detected as early as 1 DPI. The cell count increased significantly at 7 DPI, peaking at 14 DPI, declining at 28 DPI, and remaining stable until 56 DPI. In the OV-H group, the number of the inflammatory cells was higher during the late phase (14 DPI) compared with the number of the inflammatory cells in the OV-M group. The inflammatory cells peaked significantly at 28 DPI and slightly decreased at 56 DPI (Figure-2a). Table-2 presents the results of the quantitative evaluation of the overall inflammatory cell count.

**Table-2:** Quantitative inflammatory cell infiltration of biliary epitheliums in hamsters and mice with different time periods.

Groups	Infection period (Day)					

	1	2	7	14	28	56
OV-H	203.00 (179.00–237.88)	226.50 (199.25–271.75)	280.50 (189.25–350.00)	904.25 (383.00–1480.63)^C,G,J^	2934.50 (2367.00–3732.63)^∆,H,K,Q^	2403.50 (1845.50–2665.25)^E,I,L,S^
OV-M	161.50 (108.50–201.25)	268.50 (183.50–315.25)	1026.00 (786.63–1343.38)^B,F,L^	1393.75 (1067.25–1517.00)^C,G,Q^	434.25 (265.63–616.75)^∆,K,W^	225.00 (155.25–317.63)

The data reported in median with IQR. OV-H=*Opisthorchis viverrini* infected hamsters, OV-M=*Opisthorchis viverrini* infected BALB/c mice. Superscript symbols were marked for the significance between groups at *P<*0.05—i.e., Α=Infection day 1–2, Β=Infection day 1–7, Χ=Infection day 1–14, ∆=Infection day 1–28, Ε=Infection day 1–56, Φ=Infection day 2–7, Γ=Infection day 2–14, Η=Infection day 2–28, Ι=Infection day 2–56, ϑ=Infection day 7–14, Κ=Infection day 7–28, Λ=Infection day 7–56, Θ=Infection day 14–28, Σ=Infection day 14–56, Ω=Infection day 28–56 in the same group of infection, IQR=Interquartile range

### Reactive nitrogen species and nitrotyrosine protein expression in the biliary epithelium of susceptible and non-susceptible hosts

**Figure-3:**
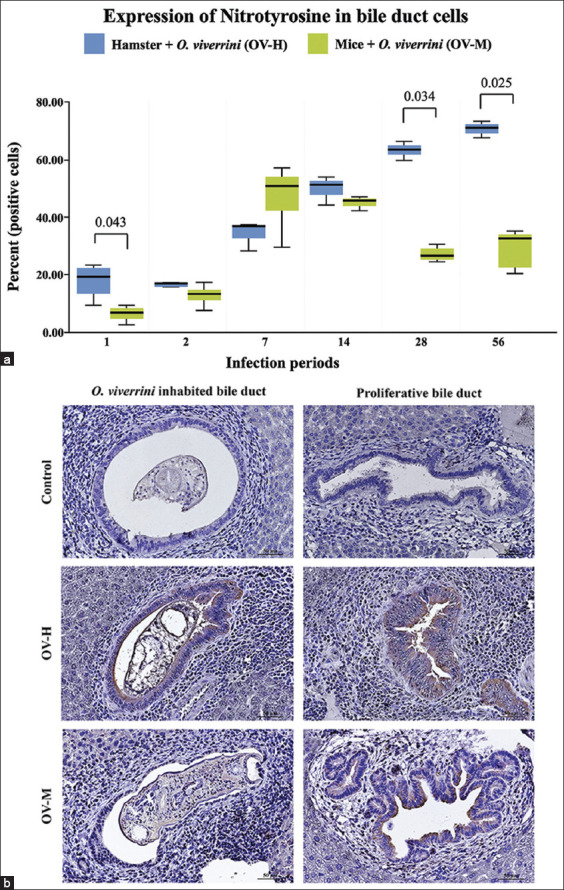
Expression in reactive oxygen species, nitrotyrosine protein in biliary epitheliums of the *Opisthorchis viverrini* infected-hamsters and mice from day 1 to 56 post-infection. (a) percentage of intracellular nitrotyrosine proteins in hamsters and mice with a significant difference, p < 0.05. (b), representative photomicrograph depicts the different intensity of nitrotyrosine in control, *O. viverrini* infected hamster (OV-H), and *O. viverrini* infected BALB/c mouse (OV-M) at worm contacted bile duct and proliferative cholangiocyte. (Immunohistochemistry, original magnification, ×40, scale bar depicts 50 μm).

We performed IHC staining using a nitrotyrosine marker in both the hamster and mouse models to explore the impact of *O. viverrini* infection-induced inflammation on ROS production in biliary cells of susceptible and non-susceptible hosts. Nitrotyrosine-positive cells exhibited a cytoplasmic staining pattern, and they were quantified and converted to a percentage (Figure-3a). Nitrotyrosine expression was initially detected at low levels in the common and primary bile ducts at 1–2 and 1–7 DPI in the OV-M group and the OV-H group, respectively. Subsequently, the intense expression of nitrotyrosine decreased and became more prominent in the second-order bile ducts, particularly in proliferating cholangiocytes (Figure-3b). The nitrotyrosine level significantly increased throughout the late stage of infection in the OV-H group, while there was a significant decrease in biliary nitrotyrosine deposits at 14–56 DPI in the OV-M group (Figure-3a). The percentage of cells positive for nitrotyrosine expression was significantly higher in the OV-H group compared with the OV-M group. Table-3 presents the changes in nitrotyrosine expression at the predetermined time points.

**Table-3:** Comparison of the percentage of intracellular nitrotyrosine proteins in hamster and mouse models with different time periods.

Groups	Infection period (Day)					

	1	2	7	14	28	56
OV-H	19.20 (11.17–22.75)	16.60 (15.69–17.16)	32.40 (28.00–37.10)^B,F^	52.58 (45.94–54.00)^C,G^	61.54 (59.65–65.66)^∆,H^	72.04 (68.30–73.08)^E,I^
OV-M	6.62 (3.43–8.70)	13.30 (9.17–15.90)^A^	52.40 (35.80–55.60)^B,F^	46.45 (43.07–47.20)^C,G,Q,W^	26.37 (24.68–29.60)^∆,H,K^	28.21 (21.32–34.56) ^E,I,L^

The data reported in median with IQR. OV-H=*Opisthorchis viverrini* infected hamsters, OV-M=*Opisthorchis viverrini* infected BALB/c mice. Superscript declared significance between groups at *P<*0.05—i.e., Α=Infection day 1–2, Β=Infection day 1–7, Χ=Infection day 1–14, Δ=Infection day 1–28, Ε=Infection day 1–56, Φ=Infection day 2–7, Γ=Infection day 2–14, Η=Infection day 2–28, Ι=Infection day 2–56, ϑ=Infection day 7–14, Κ=Infection day 7–28, Λ=Infection day 7–56, Θ=Infection day 14–28, Σ=Infection day 14–56, Ω=Infection day 28–56 in the same group of infection, IQR=Interquartile range

### 8-oxo-dG expression in *O. viverrini*-infected susceptible and non-susceptible hosts

**Figure-4:**
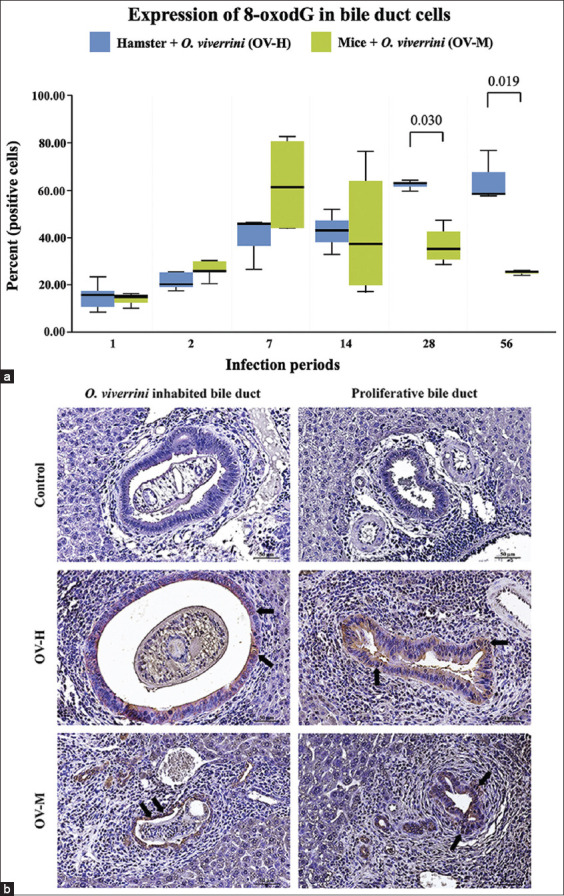
Expression of 8-hydroxy-2’-deoxyguanosine (8-oxo-dG) in biliary epitheliums of the *Opisthorchis viverrini* infected-hamsters and mice from day 1 to 56 post-infection. (a), change in percentage of intracellular 8-oxo-dG in hamsters and mice with a significant difference, p < 0.05. (b), representative micrographs of 8-oxo-dG expression intensity at *O. viverrini* inhabited bile duct and proliferative cholangiocytes in two different experimental groups. Arrow showed intranuclear staining of 8-oxo-dG marker. (Immunohistochemistry, original magnification, ×40, scale bar depicts 50 μm).

To investigate the impact of endogenous nitrosation and nitration of proteins on DNA damage, we performed IHC staining of the DNA damage marker 8-oxo-dG to evaluate its expression in biliary epithelium at predetermined time points: 1, 2, 7, 14, 28, and 56 DPI (Figure-4a). The intensity of 8-oxo-dG expression increased in both the common and primary bile ducts at 7 DPI in both experimental groups. Notably, prominent 8-oxo-dG expression was observed in proliferative secondary cholangiocytes at 7–14 DPI (Figure-4b).

Interestingly, the percentage of 8-oxo-dG-positive cells significantly increased in the OV-H group from 1 to 56 DPI, but there was no significant increase in the OV-M group (Table-4). This expression pattern correlates with the level of nitrotyrosine expression in the OV-H group. Furthermore, 8-oxo-dG expression in the OV-M group substantially decreased following a peak at 7 DPI, which corresponds with the pattern of nitrotyrosine detection (Figure-4). Table-4 presents a comparison of 8-oxo-dG results between the two animal models at the predetermined time points.

**Table-4:** Comparison of the 8-oxo-dG expression in biliary cell in hamster and mouse models with different time-periods.

Groups	Infection period (Day)					

	1	2	7	14	28	56
OV-H	16.75 (9.71–20.57)	22.29 (18.35–25.49)	36.34 (26.89–46.11)^B,F^	42.86 (35.32–49.47)^C,G^	63.60 (60.35–64.00)^∆,H^	58.29 (57.62–72.02)^E,I^
OV-M	14.72 (11.01–15.81)	27.68 (22.85–29.90)^A^	61.34 (43.72–81.97)^B,F^	37.03 (18.27–70.36)^C^	35.01 (29.34–44.99)^∆,W^	25.63 (24.39–25.68)^E,L^

The data reported in median with IQR. OV-H=*Opisthorchis viverrini* infected hamsters, OV-M=*Opisthorchis viverrini* infected BALB/c mice. 8-oxo-dG=8-hydroxy-2ʹ-deoxyguanosine. Superscript declared significance between groups at *P<*0.05—i.e, Α=Infection day 1–2, Β=Infection day 1–7, Χ=Infection day 1–14, Δ=Infection day 1–28, Ε=Infection day 1–56, Φ=Infection day 2–7, Γ=Infection day 2–14, Η=Infection day 2–28, Ι=Infection day 2–56, ϑ=Infection day 7–14, Κ=Infection day 7–28, Λ=Infection day 7–56, Θ=Infection day 14–28, Σ=Infection day 14–56, Ω=Infection day 28–56 in the same group of infection, IQR=Interquartile range

## Discussion

Chronic inflammation induced by *O. viverrini* is an important risk factor for CCA development in humans residing in countries along the Greater Mekong Subregion, particularly Northeastern Thailand [1]. Only a small portion (5%) of susceptible individuals infected with this parasitic helminth develops malignancy [4]. CCA development is believed to involve various inflammatory responses, particularly in the context of chronic inflammation, and CCA susceptibility is thought to be driven by oxidative stress and DNA damage resulting from severe and prolonged inflammation [12, 16]. Due to the limited availability of human experiments, these mechanisms are investigated using animal models.

Our study examined biliary cells in the intrahepatic parenchyma and CBD of 30 Syrian golden hamsters and 30 BALB/c mice infected with *O. viverrini*. We consistently observed the same patterns of inflammatory cell infiltration, nitrotyrosine, and 8-oxo-dG, indicating early inflammatory responses in mice along with other markers. In contrast, the hamster model demonstrated delayed responses. Since these pathological events were consistently more pronounced in susceptible hosts than non-susceptible animals, we hypothesize that *O. viverrini* worms cannot establish residence and attain maturation in mice. Furthermore, after their expulsion, the damaged cells may have the potential for curative and reparative processes.

The pathogenesis of *O. viverrini*-induced inflammation is believed to be attributed to the somatic and excretory/secretory antigens released by this parasite, which stimulate and recruit inflammatory cells to the bile duct, where they reside. In addition, chemoattractant molecules and damage-associated molecular patterns along with microbial bacteria enhance or exert a synergistic effect on the attraction of inflammatory cells [24–26]. In hamster studies on the response to helminth infection, inflammatory cells, such as neutrophils, mononuclear cells, eosinophils, and mast cells, have been reported as common white blood cells [12, 18, 21, 27]. In addition, *in vivo* experiments have demonstrated high levels of infiltration by these cell populations during the first 1–2-month post-infection [18, 27], while a study on mice reported a lesser effect [11]. Another histopathological study on mice reported persistent periductal inflammation 23 days after the initial infection [28]. Our study findings are consistent with these earlier reports and also provide additional detailed information across different time intervals.

Inflammatory cell infiltration in the hamster model was significantly higher than in the mouse model, particularly at 28 and 56 DPI, whereas a significantly higher number of cells were observed in the mouse model at 7 DPI. This suggests that a prolonged, chronic infection occurs in the hamster model. The decline in inflammation can be attributed to worm elimination. In other non-susceptible rodent models of opisthorchiasis, such as mice or rats, the worm cannot reach maturity and produce eggs [11]. Our unpublished data suggest the elimination of *O. viverrini* in the mouse model because no parasite eggs were detected, and sections of worm were only observed up to 28 DPI.

Studies on the differences in inflammatory response between susceptible and non-susceptible hosts following *O. viverrini* infection are scarce. Our study revealed that BALB/c mice exhibited better and stronger regulation of the immune response in the biliary tree during the early phase of infection. This may be associated with the peak production of Th2 cytokines, particularly interleukin (IL)-5 and IL-10, at 14 DPI [29]. The increased IL-5 production is likely related to eosinophil-mediated destruction of *O. viverrini*, supported by reports of eosinophils killing *Schistosoma mansoni* through antibody-dependent cell-mediated cytotoxicity mechanisms [30]. IL-10 induction enhances Th2 differentiation and downregulates Th1 cytokine responses, thereby promoting worm expulsion in various helminth infections [31–33]. Interestingly, our hamster model exhibited opposite responses, with the inflammatory process persistently increasing during the late phase of infection. We speculate that the susceptibility in this model may be related to a higher level of IL-4, which was reported to peak strongly around 28 DPI in susceptible mice infected with *Clonorchis sinensis* [29]. Similarly, Jittimanee *et al*. [34] reported higher levels of IL-4 and transforming growth factor-βeta in hamsters with opisthorchiasis at the same time point. The induction of both cytokines indicates that immunosuppressive functions have allowed parasites to evade the host immune response and establish a life-long chronic infection [34–36]. However, further investigations are necessary to explore the individual profiles of leukocyte responses in susceptible and non-susceptible hosts.

The pathological impact of *O. viverrini* infection through inflammatory responses is significant and can exacerbate pathology and potentially initiate carcinogenesis [37]. The infection causes increased chronic biliary inflammation that promotes inflammation-mediated DNA damage by generating free radicals [12, 37]. Upon activation by inflammation, we detected nitrotyrosine at 1 DPI in both models. However, the percentage of nitrotyrosine in mice peaked at 7 DPI and was higher than in hamsters. Considering the initial inflammation peak at 7 DPI and the later peak at 14 DPI, this pattern of nitrotyrosine production in mice may be associated with the acute response of the innate immune system, particularly resident macrophages in the submucosa. A significantly higher number of macrophages in the lamina propria have been reported during the early stages of *O. viverrini* infection in hamsters [12]. In this stage, macrophages primarily contribute to producing free radicals, which act as cytotoxic effectors, promote tissue injury, and damage DNA [38–40]. Our data further explains the relationship between free radical production and DNA damage in susceptible and non-susceptible animals. The level of 8-oxo-dG, a marker of DNA damage, was higher in the mouse model compared with the hamster model at 2, 7, and 14 DPI and peaked at 7 DPI in mice. This suggests that the damage may be more severe and induce the death of cholangiocytes in mice. In contrast, the DNA damage in hamsters may be less severe but persistent, leading to accumulated genomic instability and increased errors during DNA synthesis and repair. This, in turn, serves as a driver for tumorigenesis [41]. These findings significantly contribute to understanding the underlying mechanisms linking the inflammatory process to DNA damage and the development of severe pathology and CCA in susceptible hosts.

## Conclusion

This is the first study to demonstrate differences in inflammatory responses, nitrative free radical production, and DNA damage in susceptible and non-susceptible hosts exposed to the carcinogenic liver fluke *O. viverrini*. Our findings show that susceptible hosts exhibit prolonged immune responses that promote biliary DNA damage, suggesting that this pathogenic helminth may contribute to the development of biliary malignancy through persistent DNA damage, particularly in susceptible hosts. Conversely, non-susceptible hosts may be less affected by *O. viverrini* due to the shorter lifespan of this parasite in the hepatobiliary system. Our results provide valuable insights for further detailed investigations of the immunopathology and molecular pathways involved in carcinogenesis.

## Authors’ Contributions

ST: Conceptualization and investigation. NT, PT, RC, TT, and ST: Methodology. WS, ST: Validation and writing–review and editing. WS: Formal analysis and writing–original draft preparation. PT and ST: Supervision and editing. All authors have read, reviewed, and approved the final manuscript.
